# Analyzing Qualitative Changes in Metalinguistic Processing in Typically Developing 5- to 7-Year-Old Children

**DOI:** 10.3390/brainsci13101447

**Published:** 2023-10-11

**Authors:** Sergio Melogno, Maria Antonietta Pinto, Marco Lauriola

**Affiliations:** 1 Department of Psychology of Development and Socialization Processes, “Sapienza” University of Rome, 00185 Rome, Italy; mariantonietta.pinto@uniroma1.it (M.A.P.); marco.lauriola@uniroma1.it (M.L.); 2 Faculty of Psychology, “Niccolò Cusano” University of Rome, 00166 Rome, Italy

**Keywords:** metalinguistic development, metalinguistic tasks, cognitive levels, generalized estimating equation analysis, qualitative analysis, developmental trajectories

## Abstract

This study was based on an analysis of some of the qualitative aspects underlying the findings of previous research into the metalinguistic abilities in 160 Italian-speaking, typically developing children aged from 5 to 7 years. This previous research had used six metalinguistic tasks, a nonverbal intelligence test, and two lexical- and grammar-comprehension tests. The outcomes showed a significant improvement in all the dependent variables in the age range considered, measured by a series of ANOVAs, with high correlation between all the variables and a strong homogeneity between the metalinguistic tasks, as revealed by a factor analysis. Using generalized estimating equation (GEE) analysis, the current study analyzed the cognitive levels of response that constituted the total score of each task, at each age (5–6, 6–7, and 7–8 years). Although based on the different distribution of the cognitive levels at each age and in each task, the results of this analysis further confirmed the significance of the developmental changes, and showed different developmental trajectories as a function of the specific task. These results are discussed in light of the different involvement of cognitive processes and literacy skills in the transitional phase between kindergarten and the first two years of primary school.

## 1. Introduction

The present study drew on the outcomes of a previously published study on metalinguistic development in children aged from 5 to 7 years [1] by exploring, in more depth, some qualitative aspects that had been deemed relevant. By metalinguistic development we mean the development of the ability to reflect upon forms, meanings, and, generally speaking, structures of language beyond basic comprehension and production skills.

The field of metalinguistic awareness has long attracted the attention of scholars of different language domains, from philosophers of language [2], linguists [3,4], and applied linguists [5,6,7], to a host of developmental psycholinguists. Starting in the 1970s, a few studies on single areas of metalinguistic development were published [8,9,10,11,12,13,14] and these were later synthetized into systematic reviews during the 1980s [15,16] and the beginning of the 1990s [17]). Interestingly, both Tunmer et al. and Gombert [16,17] indicated the ages of six and seven as a turning point in the way children handle the contents and the directions of metalinguistic tasks, and both authors interpreted this turn as a reflection of emerging metacognitive processes which could also be observed in nonlinguistic domains.

The variety of disciplines that have shown interest in metalinguistics explains the multitude of terms that attempt to define the very construct of metalinguistics, with each offering a different angle on the concept. Simard and Gutierrez [18] recently presented an overview of the main terminological variations of the issue. For instance, for certain authors, of which Ellis [19] (2004) is an emblematic representative, the expression “metalinguistic knowledge” stands for declarative knowledge about specific language domains (lexicon, grammar, phonology, etc.), each coded by a specific metalanguage. Other authors focused on the attentional factors by which the speaker distances himself from language, analyzes its structural features, and treats these features as objects of thought [16,20]. The expression for these phenomena is “metalinguistic awareness”. Authors like Bonnet, Tamine, and Gardes [21]; Gombert [17]; and Simard, Guénette, and Bergeron [22] referred to the same phenomena as a manifestation of “metalinguistic reflection and activity”. Contrary to this generalizing approach, Bialystok [23,24] focused on specific “metalinguistic abilities” or “metalinguistic skills” as measured by specific metalinguistic *tasks*, defined on the basis of two components—linguistic analysis and control—whose dominance may vary according to precise contextual requests. In our work we refer to both the general construct of metalinguistic awareness, meant as the ability to consider and objectify the structural features of language, and domain-specific metalinguistic abilities, meant as single manifestations of an underlying metalinguistic awareness.

In parallel with this interest in definitions, psycholinguists (particularly Bialystok [23,24] and Karmiloff-Smith [25]) have also attempted to model metalinguistic development. Many studies have also analyzed the relationship between single aspects of metalinguistic development and literacy development (see [26,27] for a review), bilingualism (see the meta-analyses of Adesope et al. [28] and Ware, Kirkowski, and Lum [29]), and various cognitive aspects, particularly executive function (see the review of Andrade Gadelh [30]). 

Our previous study was carried out with native Italian monolingual, typically developing children using a battery of six Italian metalinguistic tasks, a grammar- and a lexical-comprehension test [31], and a nonverbal intelligence test [32]. The results highlighted a significant increase in the means in all the performances, year after year, as shown by a series of analyses of variance (ANOVAs), high correlations between all the measures, and strong homogeneity between the metalinguistic tasks, as revealed by a factor analysis where a single factor could account for a large part of the common variance. 

The aim of the present study was to analyze *the nature of the qualitative changes underlying the overall growth in metalinguistic abilities* in the age ranges considered. To this end, we broke down the total score obtained in each metalinguistic task into *the qualitative levels that were constitutive* of the score itself. This was because the coding system of these tasks did not rely on a simple dichotomic criterion (a correct versus an incorrect response), but on three levels of response of growing cognitive complexity. The cognitive nature of these levels of response lay in the fact that each represented a way of facing the metalinguistic conflict posed by each task. The child might more or less elude the conflict in question or offer a partial resolution of it or else, at the best level, propose an entirely satisfactory resolution of this conflict. For instance, in the *symbol-substitution* task [33], children were asked to replace a word with another in a given sentence, which led to a new, anomalous sentence. For example, if the child is given the sentence “The cats are under the tree” and told to pretend that ‘under’ is called ‘play’ and then asked what the sentence would be, the expected response would be “The cats are play the tree”. The replacement required clearly creates a semantic and grammatical conflict, and it is precisely this conflict that is at the core of the metalinguistic problem posed by the task. To solve it, the child must operate at an abstract level *for the sake of the conventionality principle* per se, as the basis of all verbal signs, however unacceptable the resulting sentence could be. What is observed is that children elaborate this type of semantic and grammatical conflict at very different cognitive levels. They might simply ignore the conflict and repeat the stimulus sentence, remain silent, change and/or add some words to make the new sentence acceptable (e.g., “The cats play under the tree”), or change precisely the words required, beyond the evident unacceptability of the resulting sentence (“The cats are play the tree”).

The novelty of the current study was precisely in the analysis of how these qualitative levels of response evolved in each task in the age range considered, between 5 and 7 years. This is because this type of progression by levels reveals significant changes in the very way children distance themselves from current language usages and express their position in relation to these usages. In other words, these changes are revealing of an emerging metalinguistic modus operandi, that allows a more abstract way of treating an essential instrument of thought, such as language. At the beginning of primary schooling, this, in turn, is of the utmost interest to the teacher, as well as to the developmental psycholinguist, the school, and the clinical psychologist.

We expected to observe a shift with age from the poorest level which consists of a total elusion of the metalinguistic conflict posed by each task, to the intermediate level, which shows a partial resolution of the conflict, to the highest level, which shows a satisfactory resolution of this same conflict. Actually, these three levels are presumably present at each age but in different proportions, and *what changes is the relative distribution year by year*, with an expected dominance of the lowest level in the youngest group (5–6 years) and a gradual decrease at the subsequent ages (6–7 years and 7–8 years), replaced by an increasing presence of the intermediate and highest levels.

## 2. Materials and Methods

### 2.1. Participants

One hundred and sixty children (N = 160) from 5 to 7 years old participated in this study (age range: 5 years and 0 months to 7 years and 11 months (M = 76.75 months; SD = 10.01 months, clustered into three age groups: (1) 5 years and 0 months to 5 years, 11 months, and 30 days (referred to as 5–6 years); (2) 6 years and 0 months to 6 years, 11 months, and 30 days (referred to as 6–7 years); and (3) 7 years and 0 months to 7 years, 11 months, and 30 days (referred to as 7–8 years). The sample was gender-balanced: 73 girls and 85 boys, and two participants had missing gender information.

### 2.2. Instruments

The metalinguistic tasks used in this study were:

Word order (W.O., from Ricciardelli, Rump, and Proske [34] rearranged from Pratt, Tunmer, and Bowey [35]), assesses children’s awareness of word-order rules. The task presents nine sentences where canonical word order is upset by an increasing number of displacements. Conflict is generated by these displacements, and children must recompose the sentence by re-establishing the correct word order. For example, “*Bananas are blue not*” (expected response: “Bananas are not blue”). Scoring. 0: the child does not respond or simply repeats the item or transforms the meaning of the item in order to make it semantically plausible but with no intervention on the wrong word order (e.g., “Bananas are yellow”); 1: the child does operate on word order but also makes unrequested substitutions or additions of words (e.g., “Bananas and apples are not blue”) or implements only one of the relevant changes when the item requires more than one (e.g., “The bird in the tree is”); 2: the child makes all the required transformations to re-establish the correct word order. Maximum score: 18.

Lexical segmentation (L.S., from Pontecorvo, Tonucci, and Zucchermaglio, [36]) assesses children’s awareness that linguistic units are identifiable within a given utterance by means of precise boundaries. The task presents eight increasingly longer sentences, and the child is asked to identify and count the linguistic unities in each sentence. Conflict is potentially present every time one must identify the initial and final part of each word. The examiner reads the sentences aloud but the child is allowed to look at the printed form of these sentences on a sheet of paper. For example, “The house is red” (word count: four) or “We drink coffee and milk for breakfast” (word count: seven). Scoring. 0: the child wrongly segments a single word into two parts (“break” and “fast” in breakfast”) or unites articles or prepositions with words (“wedrink” instead of “we” and “drink”); 1: the child segments nearly all the unities correctly but still makes some errors, which have negative consequences on the word count, e.g., “We drink coffee and milk for break/fast” (six unities instead of five) or the child identifies each linguistic unity correctly but fails in the total number of words); 2: responses are completely adequate on both sides—identification and counting. Maximum score: 16.

Word length (W.L., from Ricciardelli, Rump, and Proske [34], rearranged from Papandropolou and Sinclair [12], and Berthoud-Papandropolou [13]) assesses children’s awareness of the different nature of form and content in words. The task presents 10 items with words of different length, and the child is asked to say if the word is long or short. In some items, the conflict arises from the contrast between the length of the word (long or short) and the size of the corresponding referent (big or little). For example, “Train: is it a long or a short word?” (expected response: “short”) or “Strawberry: is it a long or a short word?” (expected response: “long”). In other items, the word (long or short) has no precise referent so that there is no particular conflict between form and content although the child is stimulated to reflect on form per se. For example, “Interesting: is it a long or a short word?” (expected response: “long”). Scoring: For items with no precise referent, the scoring is 0/1, due to the lack of conflict between form and content. By contrast, when this conflict exists, there are two possibilities, 0 or 2, with the higher score for the correct response because the request is more demanding. Maximum score: 13.

Rhyme test (R.T., from Pontecorvo, Tonucci, and Zucchermaglio [36]) assesses children’s awareness of the difference between semantic and phonetic associations. The task presents eight items and children are asked to choose one word from a couple of words and associate it with a triad of other words on a purely phonetic basis. The conflict arises from the contrast between these two types of associations, which children must keep totally separated. For example, “If I say: ‘pine’, ‘fine’, ‘line’, what word fits better with these words: ‘wine’ or ‘tree’?” (expected response: ‘wine’). Scoring. 0: the child’s response is based on meaning instead of form (e.g., for the above item: “tree”, semantically associated with the word “pine”); 1: the response is apparently correct but the justification is based on a concrete argument instead of a phonetic criterion (e.g., for the above item: “wine, because you can drink it”); 2: the child associates words on purely phonetic grounds, and thus clearly separates form from meaning. Maximum score: 16. 

Symbol substitution (S.S., from Ricciardelli, Rump, and Proske [34], rearranged from Ben Zeev [33]) assesses children’s awareness of the arbitrariness/conventionality of the associations between form and content. The task presents 10 items, and children are asked to replace a given word with another in a well-formed sentence, which makes this sentence anomalous. Contrary to the word-order task, where the request is to correct ill-formed sentences, in symbol substitution the request is to upset well-formed sentences in the name of an abstract principle per se, namely arbitrariness and conventionality in form/content matching. The conflict arises precisely from the application of this principle, which generates grammatical and semantic violations. For example, for the sentence “She swims well”, children are asked to pretend that ‘she’ is called ‘fishes’ (expected response: “Fishes swims well”). Scoring. 0: the child does not respond at all or simply repeats the item or declares that the ill-formed sentence is unacceptable (in some cases, the child might transform the sentence by changing some words or even adding new ones in such a way as to give the sentence an acceptable aspect); 1: the child substitutes the proper word but in a different place of the sentence, again to make the sentence acceptable, e.g., for the item: “The cats are under the tree”, “under” must be replaced by “play” (expected response: “The cats are play the tree”) but the child says: “The cats play under the tree”; 2: the child implements all the correct substitutions. Maximum score: 20. 

Printed words, letters, and number identification (P.W.L.N.I, from Ricciardelli, Rump, and Proske [34], rearranged from Watson [37]) assesses children’s awareness of the category and function of distinct types of signs. The task presents nine stripes with single letters, monosyllabic and multisyllabic words, one-digit numbers or multiple-digit numbers, drawings, and complete sentences. The child is asked to indicate linguistic units of different sizes, which change from item to item. For example, stripe number 3 presents one capital letter (K), one lower-case letter (*p*), one two-digit number (37), one three digit-number (464), and one three-letter word (“dad”). The direction is: “Make a circle around the first letter of each word in this stripe” (expected response: the child must circle the letter ‘d’ of the only word present in the stripe, which is ‘dad’). The conflict has a double character—between heterogeneous categories of signs, and within the same category of the sign. Scoring. 0: the child circles several or all the units of each stripe, without distinction between letters, numbers, and words; 1: the child circles linguistic units of inappropriate size, e.g., he circles the entire word instead of the first letter or vice versa; 2: the child applies the appropriate distinction between first letter and first word, according to the request of each item. Maximum score: 18.

To sum up, our metalinguistic tasks can be considered from both a linguistic and a psycholinguistic point of view. On the one hand, each task draws on a universal linguistic principle, and, on the other, the items and the directions are conceived in such a way as to pose a cognitive conflict. For instance, the first task (*word order*) relies on a basic syntactic principle, according to which, words cannot be combined randomly. Task 2 (*lexical segmentation*) relies on the discreteness principle, according to which words have clear-cut boundaries which identify them as separate linguistic units. Tasks 3 and 4 (*word length and rhyme test*) rely on the arbitrariness principle, according to which form and content are matched on a totally conventional basis. The same applies to task 5 (*symbol substitution*). Lastly, task 6 (*printed words, letters, and number identification*) relies on the distinction between linguistic and nonlinguistic signs. However, beyond their apparent diversity, each task fundamentally relies on the *capability to rethink the relationships between form and content.*

In addition, to face the type of cognitive conflicts posed by the nature of the items and the directions of each task, children must interpret the examiner’s communicative intentions, and, at the same time, activate a series of cognitive, *non-metalinguistic* functions such as working memory, inhibition, interference control, and switching. For instance, the *word-length* task (number 3) requires that the children keep the target word in their working memory, and inhibit the representation of the content to focus attention on its form so as to control for possible interferences. The involvement of all these cognitive functions in solving a specific metalinguistic issue gives the task its psycholinguistic character.

### 2.3. Procedures

The tests were administered by three trained psychologists, following the standard oral and individual procedure. The administration took place in a quiet room of each school involved in the study, and was divided into two sessions of 30 min each. See [1] for details.

### 2.4. Data Analysis

Children’s responses for each task were categorized into total elusion, partial resolution, and satisfactory resolution of the metalinguistic conflict (using codes 0, 1, and 2, respectively). These responses were then analyzed based on the three age groups outlined in Section 2.1 (see Participants). To evaluate the statistical significance of the age-related changes from total elusion to satisfactory resolution, while considering the potential interdependence of children’s responses within each task, generalized estimating equation (GEE) analysis was applied to the tabulated data.

This approach corrects the conventional chi-square obtained from contingency tables by factoring out intra-individual differences and treating them as contributors to error variance. Because of the ordinal nature of the response categories, the model used the cumulative logit to link function to the dependent variable values, sorted in ascending order. Furthermore, the model assumed an exchangeable correlation structure, meaning that the correlations between repeated measures (i.e., the items of each metalinguistic task) within the same subject were considered equal. Complete resolution of the metalinguistic conflict (coded 2) was the reference category used for the dependent variable, while the youngest age group (5–6 years) was the reference category used for the independent variable.

## 3. Results

**Figure 1:**
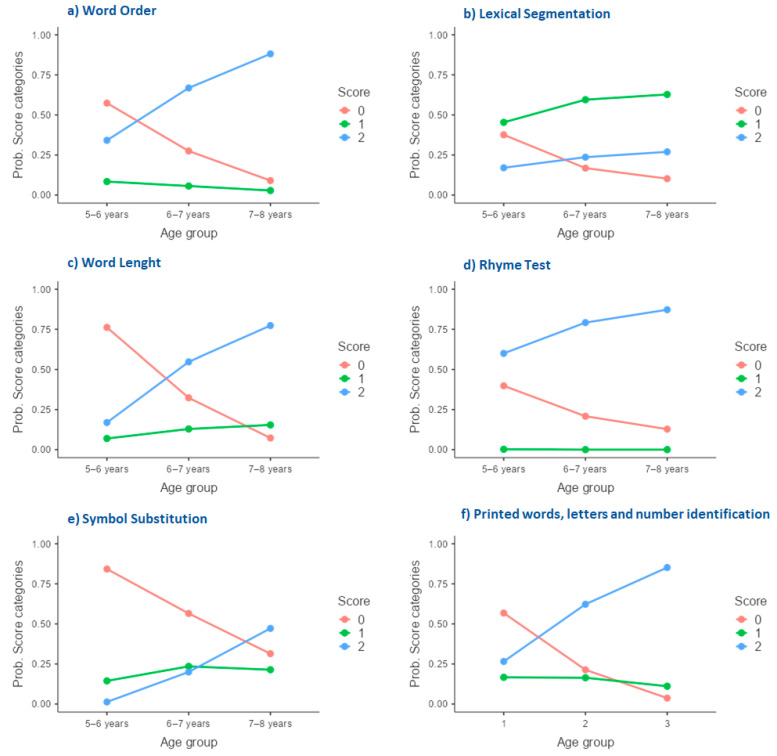
Age-related transitions in children’s responses to metalinguistic tasks.

Figure 1 illustrates the progression of children’s responses, categorized as total elusion (code 0), partial resolution (code 1), and satisfactory resolution (code 2) of the metalinguistic conflict across three age groups (5–6 years, 6–7 years, and 7–8 years).

The model predicting *word-order* response categories (displayed in Figure 1a) was overall statistically significant (chi^2^ = 49.86; df = 4; *p* < 0.001). The difference in intercepts between response category 0 and response category 2 was not statistically significant (B = 0.30, SE = 0.19, Wald = 2.61, *p* < 0.106, odds ratio = 1.35). This suggests that achieving a satisfactory resolution in the word-order task was as unlikely as complete avoidance of the conflict. Comparing the intercepts of response category 1 to those of response category 2 revealed a small but significant effect (B = 0.61, SE = 0.19, Wald = 10.63, *p* < 0.001, odds ratio = 1.84). This indicated that achieving a satisfactory resolution in the word-order task was 1.84 times more likely than achieving a partial resolution in this case. Shifting the focus to age-related comparisons, a substantial effect was noted between the 7–8 and 5–6 year age groups (B = 2.62, SE = 0.33, Wald = 62.10, *p* < 0.001, odds ratio = 13.69). The probability of a nonzero response was approximately 14 times greater for the oldest age group relative to the youngest age group (taken as the reference category). A smaller effect was noted comparing the 6–7 and 5–6 year age groups (B = 1.31, SE = 0.26, Wald = 25.22, *p* < 0.001, odds ratio = 3.70), with the intermediate age group exhibiting approximately four times greater odds of providing a nonzero response compared to the youngest age group (again taken as the reference category). Overall, the rise in response category 2 aligned with a concurrent decline in response category 0 as age increased, resulting in a crossover of response frequencies between the ages of five and seven years (see Figure 1a).

The model predicting *lexical-segmentation* response categories (displayed in Figure 1b) was barely significant (chi^2^ = 6.39; df = 4; *p* < 0.05). In comparing response category 0 to response category 2, a significant effect was found (B = −1.12, SE = 0.12, Wald = 94.80, *p* < 0.001, odds ratio = 0.33). On average, achieving a satisfactory resolution in the lexical-segmentation task was one-third less likely than completely avoiding the metalinguistic conflict. Similarly, when comparing response category 1 to response category 2, a significant effect was observed (B = 1.36, SE = 0.07, Wald = 351.15, *p* < 0.001, odds ratio = 3.90). This indicated that achieving a partial resolution in the task was approximately four times more likely than achieving a complete resolution. Shifting the focus to age-related comparisons, a significant difference was observed between the 7–8 and 5–6 year age groups (B = 0.26, SE = 0.09, Wald = 9.36, *p* = 0.002, odds ratio = 1.30). The effect was quite small, with the probability of a nonzero response being only 1.30 times higher for the oldest age group than for the youngest age group (taken as the reference category). Conversely, the comparison between the 6–7 and 5–6 year age groups yielded a non-significant effect (B = 0.113, SE = 0.0878, Wald = 1.655, *p* = 0.198, odds ratio = 1.12), suggesting no differentiation in the measured outcome between these age groups. Overall, the small rise in response category 2 aligned with a decline in response category 0 as age increased, resulting in a crossover of response frequencies between the ages of five and seven years (see Figure 1b). However, this task was characterized by the prevalence of partial resolutions over other response categories across all ages.

For *word length*, the model was statistically significant (chi^2^ = 62.67; df = 4; *p* < 0.001). Comparing response 0 to response 2 showed a significant positive effect (B = 1.22, SE = 0.25, Wald = 24.29, *p* < 0.001, odds ratio = 3.39). This means that achieving a satisfactory resolution in the task was over three times more likely than complete avoidance of the metalinguistic conflict. Contrasting response category 1 with response category 2 revealed a notable positive effect (B = 1.83, SE = 0.26, Wald = 49.288, *p* < 0.001, odds ratio = 6.26). In this case, the probability of achieving a complete resolution of the task was more than six times higher than that of a partial resolution. Age differences were particularly pronounced in this task. For example, a very large effect was observed between the 7–8 and 5–6 year age groups (B = 2.91, SE = 0.31, Wald = 89.76, *p* < 0.001, odds ratio = 18.41). This result underscores a considerable distinction in the measured outcome between these age groups, with the probability of a nonzero response being about 18 times higher for the oldest age group than for the youngest age group. A smaller but significant effect was noted between the 6–7 and 5–6 year age groups (B = 2.03, SE = 0.33, Wald = 39.12, *p* < 0.001, odds ratio = 7.64), indicating a substantial differentiation in the measured outcome between these age groups. Indeed, the probability of a nonzero response was only about eight times higher for the intermediate age group than for the youngest age group (Figure 1c). The overall trend for the *word-length* task was also very similar to that observed for the *word-order* task, with a crossover of response frequencies between the ages of five and seven years (compare Figure 1a and Figure 1c).

The *rhyme-task* model was statistically significant overall (chi^2^ = 24.02; df = 4; *p* < 0.001). When comparing response category 0 to response category 2, a significant effect was observed (B = −0.40, SE = 0.16, Wald = 6.33, *p* = 0.012, odds ratio = 0.67). This finding indicates a meaningful difference in the measured outcome between the two responses, with response category 0 having lower odds than response category 2. Turning attention to age-related comparisons, a substantial effect was observed between the 7–8 and 5–6 year age groups (B = 1.52, SE = 0.31, Wald = 23.36, *p* < 0.001, odds ratio = 4.55). This outcome underscores a considerable distinction in the measured outcome between these age groups, with the odds of providing a nonzero response category being about five times higher for the oldest group compared to the youngest group. A significant effect was found when contrasting the 6–7 and 5–6 year age groups (B = 0.93, SE = 0.25, Wald = 14.21, *p* < 0.001, odds ratio = 2.54). In this case, the odds of yielding a nonzero response category were approximately three times greater for the intermediate age group when compared to the youngest group. As depicted in Figure 1d, the *rhyme* task exhibited a ceiling effect to some extent and was characterized by the absence of partial resolutions. Unlike all the other tasks, no observable crossovers in response frequencies were identified.

The analysis of *symbol substitution* was statistically significant (chi^2^ = 57.16; df = 4; *p* < 0.001). Both the comparison between response category 0 and response category 2 (B = 1.62, SE = 0.24, Wald = 43.72, *p* < 0.001, odds ratio = 5.05) and between response category 1 and response category 2 (B = 2.76, SE = 0.24, Wald = 130.22, *p* < 0.001, odds ratio = 15.80) were statistically significant. Overall, achieving a complete resolution in this task was less likely than either a partial resolution or complete avoidance. This underscores the task’s relative difficulty compared to others in the study. A large age effect was observed comparing the 7–8 and 5–6 year age groups (B = 2.61, SE = 0.29, Wald = 81.52, *p* < 0.001, odds ratio = 13.57). Indeed, the probability of a nonzero response category was about fourteen times higher for the oldest group than for the youngest group. Similarly, a significant effect was noted between the 6–7 and 5–6 year age groups (B = 1.36, SE = 0.29, Wald = 22.31, *p* < 0.001, odds ratio = 3.90). However, the effect size was smaller, with the probability of a nonzero response category being about four times higher for the intermediate age group than for the youngest group. For this task, as for the other tasks, the rise in response category 2 aligned with a decline in response category 0 as age increased. However, a unique feature of this task was that the crossover of response frequencies between the ages of six and eight years (see Figure 1e).

Lastly, we examined the *printed words*, *letters*, *and number identification* response categories, yielding positive results (chi^2^ = 44.98; df = 4; *p* < 0.001). However, when comparing response category 0 to response category 2, we did not find statistically significant differences. Similar to the word-order task, the likelihood of achieving a satisfactory resolution in this task was comparable to complete avoidance. Conversely, when contrasting response categories 1 and 2, a significant positive effect emerged (B = 1.05, SE = 0.16, Wald = 43.82, *p* < 0.001, odds ratio = 2.87). This suggests that achieving a satisfactory resolution in the word-order task was about three times more likely than achieving a partial resolution. A substantial age effect was observed between the 7–8 and 5–6 year age groups (B = 2.65, SE = 0.43, Wald = 37.24, *p* < 0.001, odds ratio = 14.20). Again, similar to *word order*, the probability of a nonzero response in this task was about 14 times higher for the oldest age group relative to the youngest age group (taken as the reference category). A significant effect was also noted between the 6–7 and 5–6 year age groups (B = 1.55, SE = 0.22, Wald = 51.53, *p* < 0.001, odds ratio = 4.71), wherein the intermediate age group exhibited approximately five times greater odds of providing a nonzero response compared to the youngest age group. As previously noted, the overall trend for response categories in the *printed words, letters, and number identification* task (see Figure 1f) matched those observed for *word order* and *word length*, with a crossover of response frequencies between the ages of five and seven years (see Figure 1a).

## 4. Discussion

In this study, we analyzed the nature of the changes in metalinguistic processing in children from the age of five, enrolled in kindergarten, to the age of seven, enrolled in the first two primary school classes. We knew from a previous study conducted with the same tasks [1] that there was a significant improvement in this age range in the ability to cope with the metalinguistic conflicts posed by each task, as revealed by a series of ANOVAs. In the current study, we broke down the total score of each task into the single cognitive levels of response at which the conflict in question could be solved. To process our data we applied generalized estimating equation (GEE) analysis. The significance of the improvement in the age range considered was further confirmed when we analyzed the *distribution of each level*, year after year, and in each task.

In general terms, *in all tasks,* the probability of finding the poorest level, which consisted of a fundamental elusion of the metalinguistic conflict at hand, reached its maximum at the youngest age and regularly decreased thereafter, in full agreement with our expectations. Thus, for the lowest level, the developmental trend steadily and uniformly decreased. The probability of finding the intermediate level, which was based on a partial resolution of the metalinguistic conflict at hand, was less pronounced at age five and tendentially increased just one year after, except for *word length* where there was a small decrease, and a further decrease at age seven. The probability of finding the best level, which was based on a complete resolution of the metalinguistic conflict was lowest at the youngest age, following our predictions, and also increased the year after but again decreased at age seven, with the exception of *symbol substitution* where we observe a totally different trend.

In summary, the fine-grained analysis of the response levels across ages confirmed the qualitative turn that we expected in the way children distance themselves from language and rethink the relationships between form and content. However, this capability, which is at the core of metalinguistic awareness, was differently modulated as a function of the specific requests posed by the tasks. An illuminating case was represented by the two opposite developmental trends that characterized *symbol substitution, on the one hand*, and *printed words*, *letters, and number identification*, *on the other.* In the former, at age five, children seemed unable to resist the conflict that arises from the disruption of acceptable sentences to fulfill a purely abstract principle. At age six, they started accepting this abstract principle and at age seven, this capability further improved. This trend cannot be interpreted as a by-product of the schooling process since it contrasts with the teaching of basic rules of grammatical and semantic compatibility. Conversely, in *printed words, letters, and number identification*, even at age five children are able to resist the possible confusion between heterogenous signs. This capability sensibly grows after the first year of primary school but this growth nearly disappears just one year later, at age seven, which means that the improvement takes place just in the transition between kindergarten and the beginning of primary. Contrary to *symbol substitution*, this outcome can be plausibly viewed as a reflection of the acquisition of basic literacy skills.

To summarize, the two tasks mentioned above, although sharing a common metalinguistic construct, are based on a different balance between general cognitive factors and specific learning factors, an outcome that contributes to better defining the processes that underlie metalinguistic development.

## 5. Conclusions

In the future, as the tasks we used in Italian have also been translated and adapted in three more Romance languages, namely Spanish, French, Portuguese, in addition to English [38,39,40,41] it would be interesting to replicate the methodology of the present study by using the same tasks in those languages with Spanish-speaking, French-speaking, Portuguese-speaking, or English-speaking children. Possible convergences and divergences resulting from these studies would surely enrich our knowledge of the processes that govern metalinguistic development in a relevant transitional phase, between the end of kindergarten and the beginning of primary school. In turn, the outcomes obtained at this stage, especially if confirmed cross-linguistically, could become a solid basis for studying the further evolution of these abilities at more advanced ages [42].

A further prospect could be to explore the specific trends of metalinguistic development in atypically developing children, such as those with neurodevelopmental disorders, as well as in other clinical populations.

## Data Availability

Not applicable.
